# Calcium Phosphate Cements Combined with Blood as a Promising Tool for the Treatment of Bone Marrow Lesions

**DOI:** 10.3390/jfb14040204

**Published:** 2023-04-07

**Authors:** Maxence Limelette, Claire De Fourmestraux, Christelle Despas, Audrey Lafragette, Joelle Veziers, Yohan Le Guennec, Gwenola Touzot-Jourde, François-Xavier Lefevre, Elise Verron, Jean-Michel Bouler, Bruno Bujoli, Olivier Gauthier

**Affiliations:** 1 CNRS, CEISAM, UMR 6230, Nantes Université, 44000 Nantes, France; 2 Graftys SA, Eiffel Park, Pôle d’activités d’Aix en Provence, 13080 Aix en Provence, France; 3 Department of Small Animal and Equine Surgery and Anesthesia, Nantes-Atlantic College of Veterinary Medicine, Food Science and Engineering (ONIRIS), 44307 Nantes, Franceaudrey.lafragette@oniris-nantes.fr (A.L.); olivier.gauthier@oniris-nantes.fr (O.G.); 4 Regenerative Medicine and Skeleton, INSERM, University Hospital (CHU), UMR 1229-RMeS, Nantes University, 44000 Nantes, France; 5 LCPME, CNRS UMR 7564, Université de Lorraine, 54800 Villers Lès Nancy, France

**Keywords:** calcium phosphate cement, blood-containing injectable bone substitute, bone marrow lesion, bone reconstruction

## Abstract

The solid phase of a commercial calcium phosphate (Graftys^®^ HBS) was combined with ovine or human blood stabilized either with sodium citrate or sodium heparin. The presence of blood delayed the setting reaction of the cement by ca. 7–15 h, depending on the nature of the blood and blood stabilizer. This phenomenon was found to be directly related to the particle size of the HBS solid phase, since prolonged grinding of the latter resulted in a shortened setting time (10–30 min). Even though ca. 10 h were necessary for the HBS blood composite to harden, its cohesion right after injection was improved when compared to the HBS reference as well as its injectability. A fibrin-based material was gradually formed in the HBS blood composite to end-up, after ca. 100 h, with a dense 3D organic network present in the intergranular space, thus affecting the microstructure of the composite. Indeed, SEM analyses of polished cross-sections showed areas of low mineral density (over 10–20 µm) spread in the whole volume of the HBS blood composite. Most importantly, when the two cement formulations were injected in the tibial subchondral cancellous bone in a bone marrow lesion ovine model, quantitative SEM analyses showed a highly significant difference between the HBS reference versus its analogue combined with blood. After a 4-month implantation, histological analyses clearly showed that the HBS blood composite underwent high resorption (remaining cement: ca. 13.1 ± 7.3%) and new bone formation (newly formed bone: 41.8 ± 14.7%). This was in sharp contrast with the case of the HBS reference for which a low resorption rate was observed (remaining cement: 79.0 ± 6.9%; newly formed bone: 8.6 ± 4.8%). This study suggested that the particular microstructure, induced by the use of blood as the HBS liquid phase, favored quicker colonization of the implant and acceleration of its replacement by newly formed bone. For this reason, the HBS blood composite might be worth considering as a potentially suitable material for subchondroplasty.

## 1. Introduction

Since the first reports made in the 1980s [1,2,3], the use of calcium phosphate cements (CPCs) as bone substitutes in clinics is steadily expanding, in particular in trauma surgery [4,5,6,7], since they have proven their excellent ability to be replaced by new bone in a few months. However, to extend their use to a larger variety of clinical applications, many developments are currently still needed to improve, among other things, their injectability, cohesion, mechanical properties, as well as resorption rate. For that purpose, many types of additives have been added to CPCs, either to the solid or liquid phase, including for example other polymers (e.g., sodium alginate, hyaluronic acid, collagen, polypeptides, chitosan, cellulose ethers or synthetic self-assembling hydrogels) [8,9,10,11] or blood derivatives (i.e., whole blood, fresh plasma, platelet rich plasma (PRP) [12], and fibrin-based components) [12,13,14].

In this context, we have recently reported that full replacement of the liquid phase of CPCs by blood can in some cases strongly influence the properties of the resulting composites, leading to a highly injectable and cohesive paste; this exhibited far quicker resorption and remodeling of the material by bone cells when implanted in bone critical defects, when compared to the blood-free analogue [13]. In fact, two commercial formulations of apatitic calcium phosphate cements (CPCs), Graftys^®^ Quickset (QS) and Graftys^®^ HBS+ (HBS), similar in composition but with different initial setting time (7 and 15 min, respectively) were compared. Surprisingly, after full replacement of the CPC liquid phase by whole ovine blood while keeping the same liquid/powder ratio, the setting time of the HBS/blood composite was dramatically delayed when compared to its QS analogue and the two blood-free references. Moreover, the resorption rate after 12 weeks was more than twice that of the three other CPCs, with a significant replacement by newly formed bone. The promising potential of the HBS/blood composite was then confirmed using a large animal model (adult sheep), where a new approach for intervertebral spine fusion was investigated, which consisted in the injection of the HBS/blood composite in the interbody fusion cage, once placed in the intervertebral space. It was found that the fusion rate in that case was at least as good as results obtained with the classical approach, i.e., filling the cage with autologous bone graft and then insertion of the cage between two adjacent vertebrae [15].

Since these previous reports, the manufacturing process and composition of HBS has been modified leading to the marketing of a new version of this cement, i.e., Graftys^®^ HBS+. We were thus interested to investigate whether this new CPC composition had the same in vivo performances as the initial HBS. In addition, it was also important to assess the reason why this particular CPC/blood composite exhibited such an accelerated resorption rate when implanted. Indeed, the high resorption rate of the HBS/blood composite could not be accounted for by the presence of blood only, since the in vivo degradation of the QS/blood parent composite was similar to the blood-free HBS and QS references. In fact, one major difference between HBS and QS is the particle size of the solid phase. The latter was centered around 4.6 µm for QS and 19 µm for HBS, in order to tune the setting time, which was shorter when the cement solid phase was grinded more finely. In addition, although the two compositions both contained 78 wt% α-tricalcium phosphate and 10 wt% calcium-deficient hydroxyapatite (CDA) as the main components, they slightly differed for the other calcium phosphate additives.

Hence, in the present study, one batch of the HBS solid phase was divided into two parts, one of which was grinded for a longer time in order to decrease the particle size of the cement powder. Each of the two samples was then combined with blood to investigate whether the particle size of the cement solid phase influenced the physical properties of the resulting composites (i.e., setting time, cohesiveness, injectability, microstructure). Keeping in mind prerequisites for the potential medical application of such composites, commercial blood samples of human donors were used and were conditioned in sampling tubes containing classical blood stabilizers, i.e., either sodium citrate or sodium heparin. Finally, an original ovine model of a bone marrow lesion (BML) was developed, and the suitability of the HBS/blood composite to address this indication was studied in terms of surgical implantation and biological efficiency.

## 2. Materials and Methods

### 2.1. Calcium Phosphate Cements and Their Blood Composites

The apatitic calcium phosphate cements (CPCs) used in this study were obtained from Graftys SA (Aix-en-Provence, France). Graftys^®^ HBS+ (HBS) was a mixture of 78 wt% α-tricalcium phosphate (α-TCP) (Ca_3_(PO_4_)_2_), 5 wt% dicalcium phosphate dihydrate (DCPD) (CaHPO_4_, 2H_2_O), 5 wt% monocalcium monohydrate (MCPM) (Ca(H_2_PO_4_)_2_, H_2_O), 10 wt% calcium-deficient hydroxyapatite (CDA) (Ca_10−x_[]_x_(HPO_4_)_y_(PO_4_)_6−y_(OH)_2−z_[]_z_), and 2 wt% hydroxypropyl methyl cellulose (HPMC). The finely grounded Graftys^®^ HBS+ powder (HBS-g26) had the same chemical composition as HBS. Graftys^®^ Quickset (QS) was a mixture of 78 wt% α-TCP, 10 wt% anhydrous dicalcium phosphate (DCPA) (CaHPO_4_), 10 wt% CDA, and 2 wt% HPMC. The liquid phase consisted of a 5 wt% Na_2_HPO_4_ aqueous solution (liquid/powder ratio = 0.59 mL·g^−1^) and a 0.5 wt% Na_2_HPO_4_ aqueous solution (liquid/powder ratio = 0.45 mL·g^−1^), for HBS, HBS-g26, and Graftys^®^ Quickset, respectively. The cement paste samples were prepared by mixing the powders with their respective liquid phase for 2 min to ensure the homogeneity of the obtained paste. The same conditions were applied for the preparation of the corresponding blood/CPC composites, except that the liquid phase was fully replaced by ovine or human whole blood stabilized by the addition of sodium citrate (3.2 wt%) or sodium heparin (14.5 to 15.8 USP·mL^−1^). Typically, according to the HBS liquid/powder ratio, 4.72 mL of blood was added and then mixed with 8 g of HBS powder.

### 2.2. Methods

#### 2.2.1. Laser Granulometry

According to usual standard procedures for calcium phosphate powders, the particle size analyses were performed on dry powders using a laser diffraction analyzer (MasterSizer 3000, Malvern Instruments, Malvern, UK). The vibration of the hopper, and the obscuration detection were, respectively, set at 100%, and 20%.

#### 2.2.2. High Frequency Impedance

The high frequency impedance measurements were recorded, between 0.4 and 100 MHz, using an experimental setup operating at 37 °C.

From the complex impedance data, noted Z*, the dielectric permittivity (ε′, related to dipole variation) and dielectric losses (ε″ related to the motion of free charges) were computed using this setup. The initial setting time (t_i_) was defined as the time elapsed until the first significant variation of dielectric parameters which reflected the α-TCP dissolution (important increase in ε′ and strong decrease in ε″). Then, the final setting time (t_f_) was defined as the second change in the variation of ε′ and ε″, corresponding to the beginning of the ageing period (ε′ reaches a maximum value and ε″ a minimum value). Each measurement was run in duplicate, and the results are given as an average value.

#### 2.2.3. SEM Characterization

Polished cross-sections of the CPC samples (hardening time: 72 h at 37 °C) were obtained using a SM09010 cross-section polisher (JEOL, Akishima, Tokyo, Japan) by applying an argon ion beam accelerated by a voltage up to 6 kV perpendicular to the surface of each specimen (around 1.5 mm^2^) for 6 h. SEM observation of the resulting samples was performed using a 7600F field emission gun scanning electron microscope (JEOL). Images were acquired on a backscattered electron mode with an 8.2 pA beam current and accelerated voltage between 8 and 10 kV.

#### 2.2.4. Injectability, Cohesion, and Decalcification Studies

- Injectabilities were assessed using a texture analyzer (LLOYD Ametek LS5, AMETEK STC, Elancourt, France) equipped with a 5 kN sensor (5KN0634, LLOYD Ametek, AMETEK STC, Elancourt, France). The test consists in measuring the required force to extrude a cement paste from a syringe. In practice, after mixing the solid and liquid phases together in a mortar for 1 min, the cement paste was transferred into a 1.6 mm section syringe (5 mL, TERUMO, Shibuya City, Tokyo, Japan). The syringe was then placed on the texture analyzer and the experiments were started 15 min after the beginning of the mixing, as recommended by the manufacturer for the use of HBS. The displacement speed applied to the syringe plunger was set at 1 mm·s^−1^ and the force necessary for extrusion was recorded until full injection of the paste or until a maximal value of 100 N was reached. A picture of the device setup is given in the Supplementary Materials section. The extruded paste was directly collected in a 0.9 wt% sodium chloride solution placed below the syringe outlet. At the end of the extrusion, this solution was placed in an oven at 27 °C, and the cohesion of the paste right after injection and 24 h later was visually controlled. Each measurement was run in triplicate and the results are given as mean ± standard deviation (SD). The syringe was weighed empty and once filled with the cement sample. The syringe was weighed again at the end of the extrusion process, thus allowing us to determine the percentage of extruded paste for each assay.
- Once hardened for 72 h in a 0.9 wt% NaCl solution at 37 °C, the molded cylindrical samples of CPC combined with blood (6 mm in diameter and 12 mm in height) were decalcified by immersion in 30 mL of a 10 wt% aqueous solution of ethylenediaminetetraacetic acid (EDTA, Merck KGaA, Darmstadt, Germany) at room temperature for 3 weeks, with a renewal of the EDTA solution every 5 days. Then, the samples were successively submerged in a 4 wt% formaldehyde solution (5 mL, 2 days), 70% ethanol (10 mL, 1 day), 90% ethanol (10 mL, 1 day), and pure ethanol (10 mL, 8 days). The samples were then rinsed in a water/acetone mixture by increasing the acetone content progressively to end-up with a final rinsing with pure acetone.

### 2.3. In Vivo Implantation of CPC and Blood Composite CPC

#### 2.3.1. In Vivo Implantation of CPC in Vendeen Sheep Model

The animal study protocol received approval from the regional ethical committee for animal experimentation, was authorized by the French Ministry of Higher Education, Research and Innovation (Apafisnumber #11192), and was conducted in conformity with the European Guidelines for the Care and Use of Laboratory Animals (2010/63/UE). Accordingly, sheep were housed in the animal facility of the ONIRIS College of Veterinary Medicine (Agreement No. E44271). During the stalling period, animals were housed under standard conditions and checked every day. Moreover, water and food were available at will.

Six adults female Vendeen sheep (3–7 years old) with an average body weight of 81 kg were used in this study. Animal handling and surgical procedures were conducted at the Research and Preclinical Investigation Centre at ONIRIS. A 24 h fasting period was applied before surgery to decrease the volume of the rumen content.

The six animals were implanted with HBS on the one hindlimb versus its blood composite HBS + citrated ovine blood (HBS blood composite) on the contralateral one.

The HBS cement was prepared by mixing the HSB powder with a 5 wt% Na_2_HPO_4_ aqueous solution (liquid to powder ratio = 0.59). The HBS blood composite was prepared by mixing 8 g of Graftys^®^ HBS+ sterile cement powder into 4.72 mL of freshly collected stabilized (sodium citrate) autologous blood harvested from the marginal vein of the ear (no blood treatment). 

After an intravenous premedication with methadone (0.2 mg/kg), medetomidine (0.005 mg/kg), and diazepam (0.1 mg/kg), general anesthesia was induced with ketamine (5 mg/kg IV) and propofol (1–2 mg/kg IV to effect). Anesthesia was maintained for surgery with a gas mixture of sevoflurane (EtSevo 1.5–2.3%), in an oxygen/air mixture (FiO_2_ 40%), in association with a continuous infusion of lidocaine (3 mg/kg/h) and ketamine (1 mg/kg/h). Postoperative analgesia included meloxicam 0.5 mg/kg IV repeated daily for 4 days and a surgical wound infiltration subcutaneously with bupivacaine 0.5% (1 mg/kg) at the end of the procedure.

**Figure 1:**
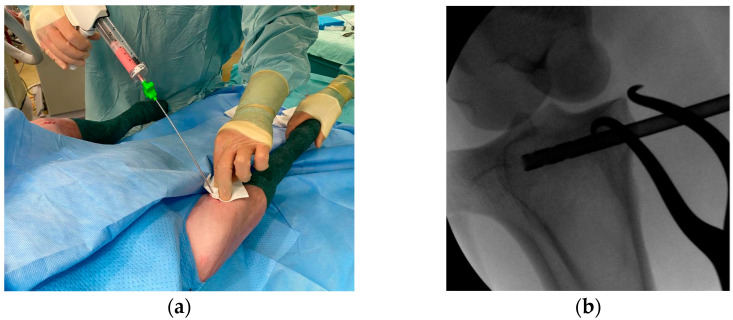
(**a**) Implantation procedure of the cement formulations (**b**) under fluoroscopic guidance in the induced bone cavity.

Briefly, with the animal in dorsal recumbency, hindlimbs were aseptically prepared for bilateral implantation. A 1 cm long cutaneous incision was made over the medial surface of the tibia a few millimeters below the stifle joint; subcutaneous tissues were incised on the same line and reclined with a mini-Gelpi self-retaining retractor. A 4 mm drill bit was used to create a bone tunnel immediately distal to the tibia plateau under fluoroscopic guidance (Figure 1b), from medial to lateral. Bone debris was removed by saline irrigation. The tested materials were then implanted bilaterally for 4 months in the induced bone tunnel. The cement injection was imaged using fluoroscopy. As reported in Figure 1a, cement was injected in a retrograde manner with an 8 G cannula to fill the tibial bone tunnel. At the end of injection, hemostasis was checked for a few minutes to be sure that the injected cement was not pushed out of the bone cavity by intramedullary hemorrhage.

Subcutaneous and skin tissues were then routinely sutured. At the end of surgery, animals received an injection of 0.5 mg/kg of meloxicam (Métacam Bovin^®^, Boehringer Ingelheim, Germany) to provide analgesia that was repeated every day during the five following postoperative days. Animals were kept in individual cages during the first two postoperative days and were allowed to move freely in collective runs thereafter. No perioperative and postoperative complications were observed. Animals were XR imaged at 1 and 12 weeks after surgery and sacrificed after 18 ± 1 weeks.

Four months after implantation, general anesthesia was induced by a mixed injection of ketamine (Imalgène^®^1000, Merial, France) and xylazine (Rompun^®^, Bayer, France) and the animals were euthanized by an intravenous injection of 240 mg/kg of pentobarbital (Doléthal^®^, Vétoquinol S.A., Lure, France) through a catheter placed into the jugular vein. The explanted bone specimens were immediately placed in a 10 wt% aqueous formaldehyde solution and stored at 4 °C before analysis, including 3D microcomputer tomodensitometry (µ-CT), scanning electron microscope (SEM), and light microscopy histological study on stained sections.

#### 2.3.2. Human Blood CPC Composite

Human-based blood CPC composites were obtained by combining human blood with the Graftys HBS+ cement powder as previously described. The human blood was purchased from Cambridge Bioscience (UK), and harvested from a 28-year-old healthy male donor. Blood was stabilized in sodium heparin or sodium citrate during the collection process, and delivered overnight at 5 °C. Blood tubes were kept at 4 °C, and a maximal period of storage of 3 weeks was routinely applied before use. IRB certificates are given in the supplementary section.

### 2.4. Data Acquisition and Analyses

#### 2.4.1. Microcomputer Tomography (µ-CT Analyses)

The tibial specimens were inserted in a foam base impregnated with alcohol, and the samples were imaged using X-ray radiation micro-CT (Skyscan 1272^®^, Bruker, Kontich, Belgium). Scanning analysis was performed at 26 μm isotropic resolution with the X-ray tube operated at 100 kV and 100 μA. Image reconstruction was performed using the NRECON^®^ software v1.7.4.6. The CTAn^®^ software v1.20.8.0+(64-bit) was used for image analysis and the DataViewer^®^ software v1.6.0.0(64-bit) (orientation, VOI selection) for 3D visualization of the scanned areas. Quantitative analysis of the bone content (Tissue volume TV, Bone volume BV, and BV/TV ratio) into a standardized region of interest (VOI) slightly larger than the bone tunnel (VOI: 225 × 225 × 750 vx; 5.85 × 5.85 × 19.5 mm) was determined using both 2D and 3D measurements. The calculation of other parameters included trabecular thickness (Tb.Th, µm), trabecular separation (Tb.Sp, µm) and trabeculae number (Tb.N). The results obtained for all parameters were compared with each other in the two conditions of implantation in the study, HBS versus HBS blood composite, and a control untreated selected bone of an equivalent VOI.

#### 2.4.2. Scanning Electron Microscopy (SEM) Analyses

The fixed bone specimens were dehydrated with graded ethanol, and were then infiltrated and embedded in glycol methyl methacrylate (GMMA). The samples were then cut into two pieces using a Leitz^®^ diamond saw, passing through the center of the tibial bone tunnel parallel to the medio-lateral drilling axis to provide longitudinal sections, and perpendicularly to the drilling axis to provide transverse sections. The resulting resin blocks were polished and then sputtered-coated with Au/Pd (Desk V^®^ Denton vacuum, Moorestown, NJ, USA). The samples were observed by SEM (GeminiSEM 300, Zeiss, Germany) and the images were acquired with the back-scattered electron detector. A full image of the implant surface was obtained by the reconstruction of the selected scanned area images (SmartStich v1.1 software, Zeiss, Jena, Germany). After an appropriate thresholding using the ImageJ Fiji software v1.54b to differentiate the newly formed bone from the implanted cement, quantitative SEM analysis was made to determine the respective amount of remaining cement into a selected area. Quantitative analyses were performed with DragonFly (ORS, Montréal, QC, Canada) version 2022.1.0.1231 using a deep learning module for realistic and accurate quantification of bone tissue. Dragonfly provides solutions for advanced image processing, segmentation, and quantification with higher accuracy compared to human experts executing the same task [16].

#### 2.4.3. Histological Analyses

In accordance with our routine protocol [17], undecalcified 7 µm-thick sections of each sample were obtained using a hard tissue microtome (HistoCore Autocut R, Leica Biosystems, Nussloch, Germany) and were then mounted on an adhesive tape (Histfilm, Watson Biolab, Kobe, Japan) to avoid stripping. Sections were observed using hematoxylin–eosin (HE) staining, Goldner’s trichrome, and Movat’s pentachrome stains. The latter was perfectly adapted to distinguish mineralized bone (yellow to green), osteoid tissue (red), and cement (blue). Quantitative histological analyses were performed to determine the proportion between bone and cement into a selected area thanks to Weka Segmentation using the ImageJ Fiji software.

#### 2.4.4. Statistical Analyses

A statistical analysis of the injectabilities and cohesions measurements was performed according to the non-parametric Kruskal–Wallis test. A *p* value less than 0.05 was considered significant.

A quantitative assessment of the remaining cement and new bone formation was performed, using a linear mixed effect model on paired measured associated to a trabecular control bone area regarding µ-CT 3D analysis. A *p* value less than 0.05 was considered significant. Variances analyses were made per individual and per tibia, when associated with a trabecular bone control.

## 3. Results & Discussions

### 3.1. Granulometry of the Studied Cement Powders

**Figure 2:**
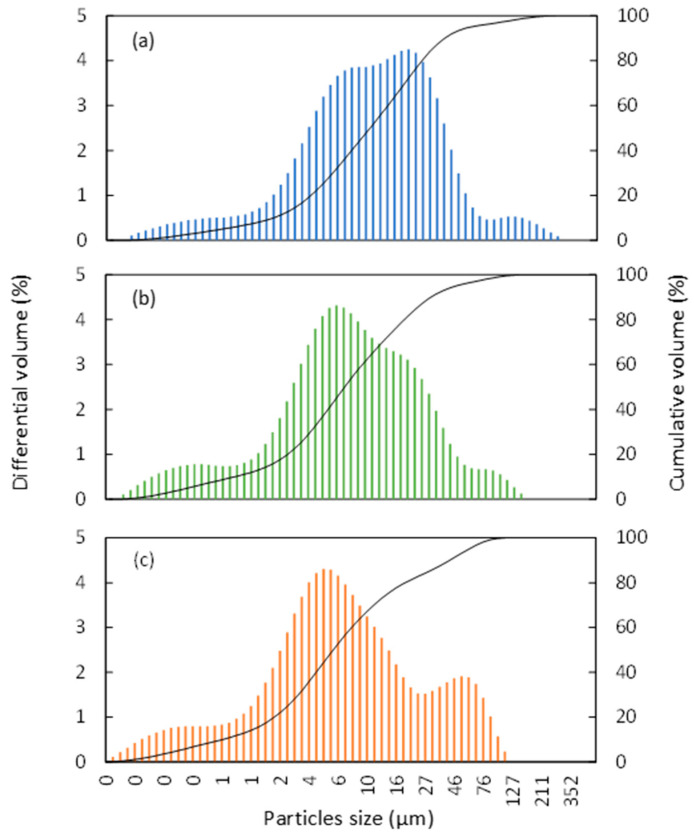
Particle size distribution obtained by laser diffraction analysis of the (**a**) HBS, (**b**) HBS-g26, and (**c**) QS powders.

The particle size of the studied cement powders was measured by laser diffraction analysis, and, as expected, the HBS powder which corresponded to the less grinded composition showed the coarsest particles (Figure 2a).

Moreover, when grinded for a longer time, the HBS-g26 samples contained a larger amount of fine particles, with a particle size distribution profile (see Table 1, and Figure 2b,c) very similar to that of the fast-setting QS CPC. Therefore, in order to investigate whether the granulometry of the CPC powder influences its physical properties once combined with blood, the HBS and HBS-g26 samples seemed suitable for this study, since they exhibited the same chemical composition but a significantly different particle size distribution.

### 3.2. Setting Time Monitoring of the Studied Cements Using High Frequency Impedance

In previous studies, we have reported that the Gillmore needles standard method [18] is inappropriate to measure the setting time of CPC/blood composites [13] by contrast with high frequency impedance measurements, which were found to be a relevant and general method for the in situ monitoring of the chemical reaction which drives the hardening process of apatitic CPCs. Indeed, the alpha-tricalcium phosphate (α-TCP) main component of the CPC is gradually dissolved with the concomitant precipitation of calcium-deficient hydroxyapatite (CDA) crystals in the intergranular space, leading to a gradual increase in the stiffness of the cement paste upon entanglement of the CDA crystals to form a three-dimensional network [19]. In fact, we have shown that the dielectric permittivity (ε′, related to dipole variation) and dielectric losses (ε″, related to the motion of free charges), which can be computed from the complex impedance data, exhibited a sharp variation at the beginning of the conversion of α-TCP into CDA [20], which denotes the initial setting time of the CPC. Then, a smooth evolution of the ε′ and ε″ values occurred corresponding to the time point at which the cement became rigid (final setting time). Finally, after a few days, the dielectric permittivity and dielectric losses started to stabilize during the CPC curing period.

**Figure 3:**
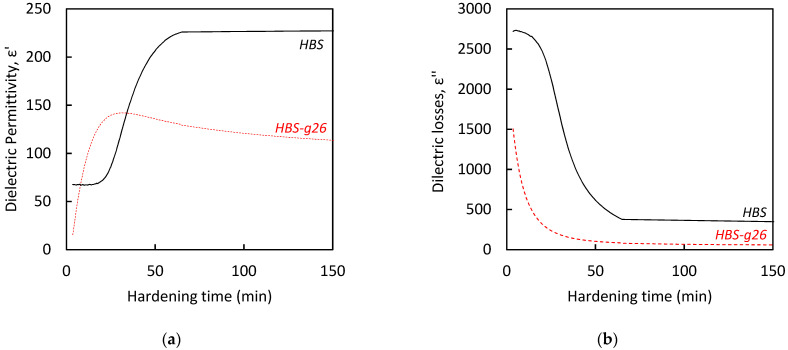
(**a**) Dielectric permittivity and (**b**) dielectric losses recorded at 37 °C during the hardening of HBS and HBS-g26 formulations.

As expected, the more finely grinded HBS powder (HBS-g26) resulted in a decrease in both initial and final setting times by comparison with the original HBS solid phase (see Table 2, and Figure 3). These values were comparable with those measured for the fast-setting Graftys^®^ QS cement [13], consistent with the fact that QS and HBS-g26 exhibited a similar particle size distribution. In the case of HBS, the larger particle size made the powder less reactive, leading to a cement composition showing slower hardening kinetics.

**Figure 4:**
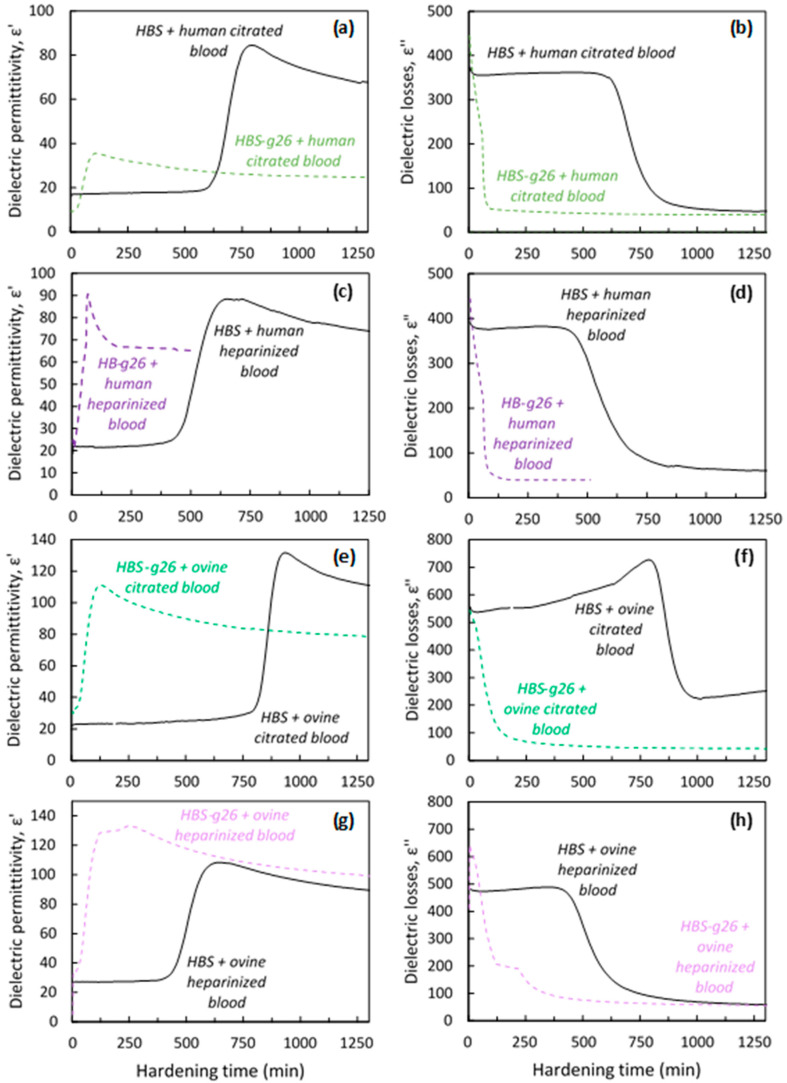
Dielectric permittivity and dielectric losses recorded at 37 °C during the hardening of HBS and HBS-g26 formulations and their analogues combined with (**a**,**b**) human citrated blood, (**c**,**d**) human heparinized blood, (**e**,**f**) ovine citrated blood, and (**g**,**h**) ovine heparinized blood.

Interestingly, a dramatic increase in the initial setting time (from 7 up to 15 h—see Figure 4) was observed for HBS when using stabilized blood as the liquid phase, depending on the nature of the blood (ovine vs. human) and stabilizer (sodium citrate vs. sodium heparin). By contrast, an increase in the initial setting time was also noticed in the case of HBS-g26 but to a far lower extent (ca. 30 min). In the case of the heparin stabilizer, the dielectric permittivity, and dielectric losses profiles for the HBS composites were the same for the two blood samples, with an initial setting time close to 7.5 h. A longer setting time was observed in the case of the citrated blood samples combined with HBS. It is important to note that the commercial human citrated blood was collected using a standard citrated tube, while for the ovine blood, a certain amount of sodium citrate was added to the tube right after the blood collection (i.e., 0.5 mL of sodium citrate to approximately 4.5 mL of whole blood leading to a final concentration of ca. 3.2 wt% sodium citrate). This might likely explain the difference of initial setting time observed for the two corresponding composites (ca. 10.5 h [human blood] vs. 15 h [ovine blood]).

### 3.3. Injectability and Cohesion Assessment of the Studied Cements

**Figure 5:**
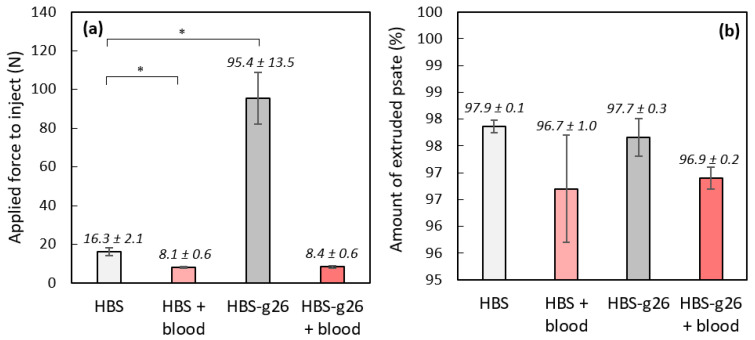
Injectability assessment for HBS and HBS-g26, and their analogue combined with ovine citrated blood: (**a**) force necessary for extrusion of the paste, 15 min after mixing the solid and liquid phases; (**b**) amount of extruded paste. (*: *p* < 0.05). Results are given as mean ± SD.

To be suitable for minimally invasive surgery, CPCs need to be fluid enough to allow easy injection, and cohesive enough to avoid disaggregation once implanted. The commercial Graftys HBS exhibited such features, and the different compositions investigated in this study were thus compared to this material of reference. Our standard procedure consisted in extruding the cement paste at a constant rate, 15 min after mixing the solid and liquid phases. As shown in Figure 5, the force necessary for extrusion of the paste was measured, as well as the amount of paste that could be injected (Figure 5b). For the four compositions, the entire content of the syringe could be injected (Figure 5b), with no statistical difference between the four conditions. However, the force necessary for extrusion of the paste was significantly higher (*p* < 0.05) for the HBS-g26 sample, since the shorter setting time of this composition resulted in a densification of the cement making it more difficult to inject. Importantly, it can be noted that the two blood-containing compositions were highly injectable, even easier than the HBS reference.

**Figure 6:**
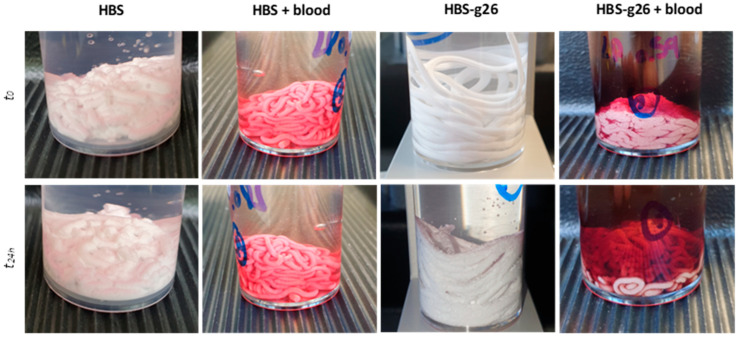
Cohesion assessment for HBS and HBS-g26, and their analogue combined with ovine citrated blood: immediately after extrusion (**top** views), and 24 h after extrusion in a solution kept at 37 °C (**bottom** views).

Moreover, while the cohesion of the HBS CPC was found to be moderate, the cohesion of the more finely grinded analogue was very good, as a result of its shorter setting time (Figure 6). Interestingly, both corresponding blood composites exhibited an excellent cohesion although their respective setting time was delayed, giving evidence of the positive role of blood which prevented disaggregation of the CPC when immersed in a liquid.

### 3.4. Analysis of the Microstructure of the Hardened Cements

**Figure 7:**
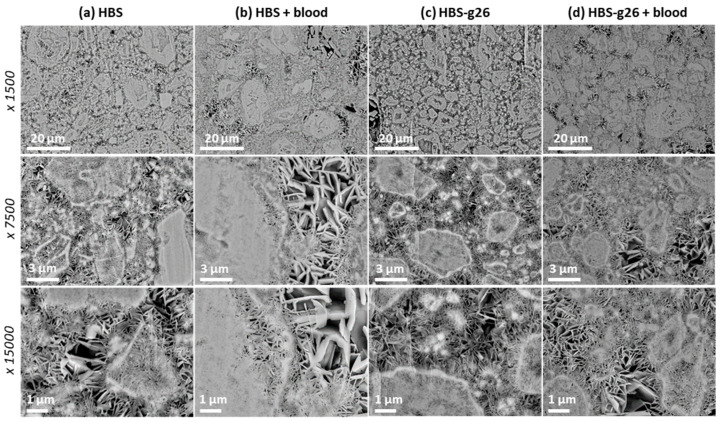
SEM observation of a polished cross-section of (**a**) HBS, (**b**) HBS-g26, (**c**) HBS combined with ovine citrated blood, and (**d**) HBS-g26 combined with ovine citrated blood after a setting time of 72 h at 37 °C. Magnification: ×1500 (**top** views), ×7500 (**middle** views), and ×15,000 (**bottom** views).

Given the marked increase in the setting time when HBS was combined with blood, this raised the question of whether the presence of blood might influence the microstructure of the corresponding composite once hardened, by comparison with its blood-free analogue. As shown in Figure 7, polished cross-sections of HBS and HBS-g26 showed a similar microstructure, in which the α-TCP particles have a geode-like shape upon transformation into CDA, homogeneously embedded in a network of small interdigitated platelet CDA crystals. As expected, the size of the geode-like particles was smaller for the more grinded HBS sample. Interestingly, in the presence of blood, the CDA platelet network between the particles for HBS showed in some places areas of lower density in which the size of the platelet crystals was significantly larger. This was also the case for the HBS-g26 blood composite, but to a far lesser extent.

To complete these data, samples of the blood composites were decalcified at different time points in order to investigate the fate of the blood component as the cement setting reaction progressed and results are summarized in Table 3. Interestingly, in the case of the HBS blood composite, no residue could be observed upon decalcification for elapsed times shorter than 24 h, but after one day, the amount of insoluble organic residues started to increase and became denser and denser in order to end up with a brick-colored solid which retained the cylindrical shape of the hardened sample before decalcification. The appearance of the insoluble organic residue started earlier in the case of the HBS-g26 blood composite. Given that the blood component can only be present in the liquid continuous phase when preparing the cement paste, blood seemed to affect the formation of the CDA crystals network between the cement particles and slowly aggregated to form a blood clot filling the space available between the CDA crystals.

### 3.5. In Vivo Implantation of CPCs and Their Blood Composites

**Figure 8:**
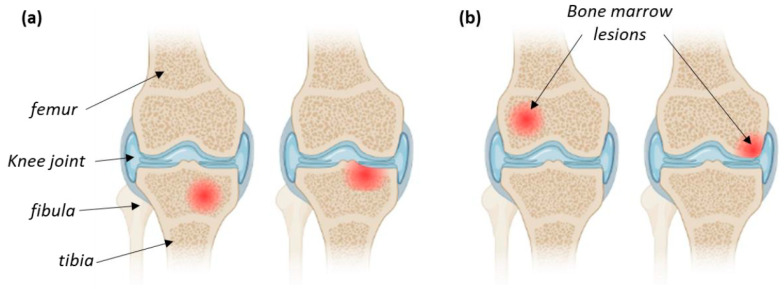
Schematic representation of different BMLs affecting (**a**) the tibia or (**b**) the femur in the knee joint area.

Bone marrow lesions (BMLs) occur in the cancellous bone tissue located at the interface with the articular cartilage in the subchondral bone area, below the articular surface or close to the ligament or tendon attachment. First reported in 1988 by Wilson et al. [21], this bone pathology results in very acute pain especially when characterized by a cyst-like lesion into the subchondral bone, most often in knee joints since this bone area usually undergoes high mechanical stresses [22]. BML are evolutive lesions whose description has been considerably improved thanks to the routine use of MRI to document joint diseases. Changes in MRI can be used to evaluate the course of the disease or the effect of a treatment [23]. As shown by Compagnoni et al. [24], BMLs can exhibit different sizes (Figure 8) and can progress toward irreversible destruction of the articular cartilage if not treated in time, thus requiring, in more severe cases, joint realignment or full joint replacement [25]. Moreover, there is a current debate on the fact that BMLs might be an early stage of more severe diseases such as arthrosis [26].

In this context, many treatments have been investigated to reduce chronic pain and stop the progress of BMLs, including (i) decompression of the bone cavity by microperforation or microfracture [27], (ii) bone resorption inhibitors in the case of osteoporotic lesions [28], or (iii) vasodilators to improve fluid circulation in the oedema region [29].

In 2007, the fluoroscopy-guided subchondroplasty technique was developed for the treatment of BMLs [30], consisting in the injection of autologous biological products to promote bone remodeling [31], i.e., platelet rich plasma (PRP) or concentrated bone marrow [27,28], although a full bone redensification was never obtained through this approach. Nowadays, the subchondroplasty technique is still proposed with, in some cases, the use of injectable CPCs for which promising clinical results were reported in both cystic-like and non-cystic-like lesions [32,33,34,35]. In this case, the use of low-diameter cannulas is needed as well as highly injectable and cohesive CPCs, given the high intra-osseous pressure needed during the injection procedure to fill the cystic-like lesion and/or diffuse into the surrounding trabecular network of the subchondral bone [36,37].

Taking account of the observed physical properties of the HBS blood composite, such a CPC might be appropriate for subchondroplasty, and therefore an original ovine BML model was developed to investigate the potential of this composite for this indication.

In an attempt to mimic the surgical treatment of a subchondral BML of the knee joint, a bone tunnel was created underneath the tibia plateau as part of the decompression procedure and as an access to allow a calcium phosphate bone cement injection [38]. Inclusion in the ongoing repair process of BML requires a highly reactive and resorbable CaP cement that can contribute to both the filling of the lesion and the rapid regeneration of the initial trabecular bone architecture.

### 3.6. Microcomputed Tomography (µ-CT) Analysis

Orientation and region of interest (VOI) selection with the DataViewer^®^ software permitted us to select a 225 × 225 × 750 vx zone, which was analyzed with the CTAn^®^ software. The quantitative analysis of the bone content (Tissue volume TV, Bone volume (BV), BV/TV (%) and trabecular parameters are summarized in Table 4. A control VOI in the surrounding trabecular bone was also analyzed and compared to both conditions. 

**Figure 9:**
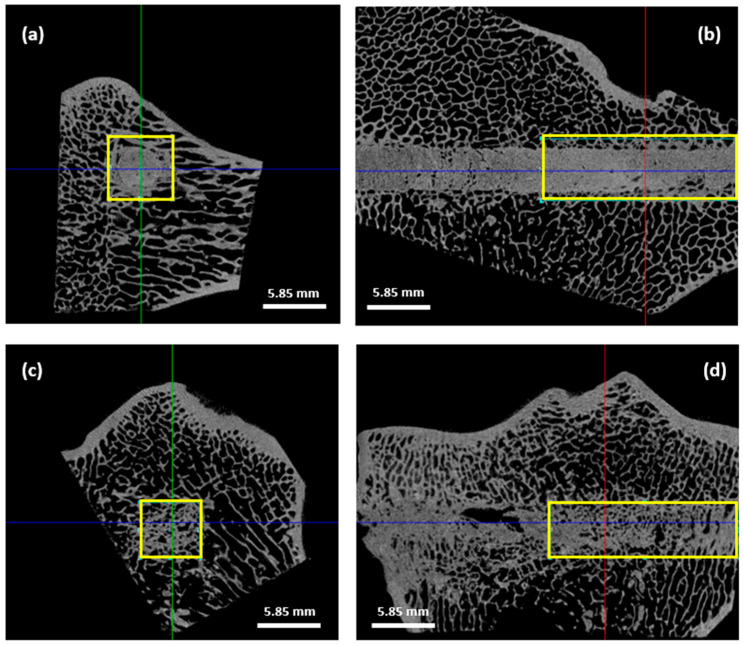
Micro-CT transverse and longitudinal images of bone specimens and selected VOI (yellow rectangle) for quantitative analyses in both HBS (**a**,**b**) and HBS blood composite (**c**,**d**) formulations. Resolution: 26 µm.

Micro-CT allowed us to distinguish both cements and to select a representative VOI for quantitative analysis (Figure 9).

The HBS cement remained dense 4 months after implantation with minimal resorption whereas the HBS blood composite showed a high level of remodeling with a noticeable induced macroporosity. Grey levels from the cements were very close to those observed in the surrounding host bone. That is the reason why results concerning tissue volume, bone volume, and the ratio BV/TV were not relevant as the CTAn^®^ software was not able to clearly differentiate bone from the implanted cements. In fact, the BV measurement included both the remaining cement and the newly formed bone inside the bone tunnel without any significant difference between the two types of cements. Although slightly lower for the HBS blood composite, thus suggesting a higher cement resorption of this cement, the BV/TV ratios were not significantly different between the two cements.

**Figure 10:**
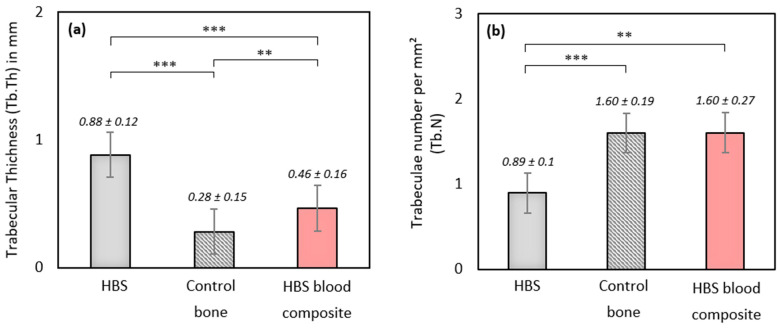
Quantitative µ-CT measurements of morphometric trabecular parameters (**a**) Tb.Th and (**b**) Tb.N after implantation of HBS vs. HBS blood composite (ovine citrate blood), and in a control untreated trabecular bone volume (**: *p* < 0.01; ***: *p* < 0.001). Results are given as mean ± SD.

More interestingly, trabecular parameters Tb.Th and Tb.N were similar between the newly formed bone associated with the HBS blood composite and the control untreated bone equivalent VOI. A significant difference in Tb.Th was observed between the two cement conditions and the control bone areas (*p* < 0.001). Tb.Th was significantly lower in the case of the HBS blood composite compared to the HBS cement (*p* < 0.01), showing that the newly formed trabeculae in the case of the implanted HBS blood composite were thinner and closer to the physiological architecture (Figure 10a). Moreover, the difference in Tb.N between the HBS blood composite and the control trabecular bone was not significantly different, emphasizing the restoration of a trabecular organization similar to the physiological one after injection of the HBS blood composite (Figure 10b). No significant difference was observed for the Tb.Sp parameter between the different conditions.

### 3.7. SEM Analyses

**Figure 11:**
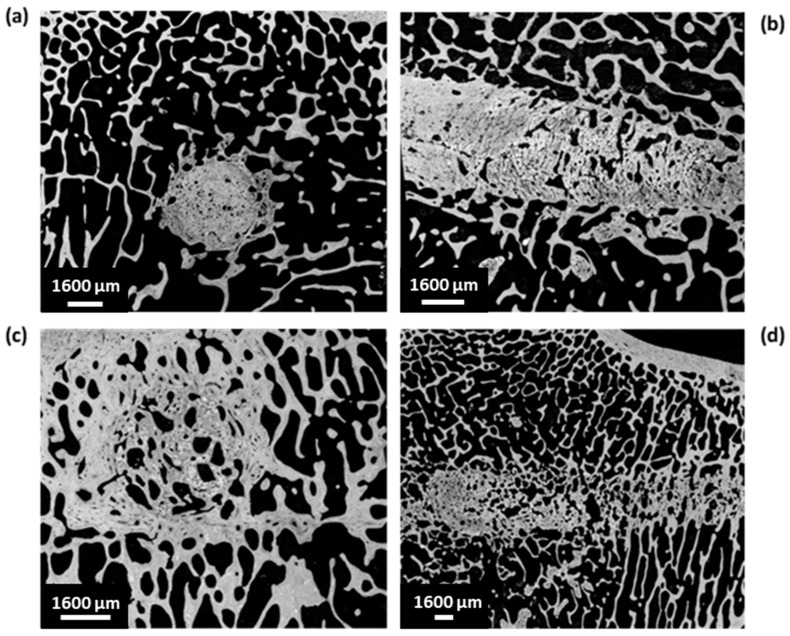
SEM images of transverse and longitudinal sections of the bone tunnel, 4 months after implantation of HBS (**a**,**b**) vs. the HBS blood composite (**c**,**d**).

Full reconstructed images of the implant surface were observed to evaluate cement distribution and new bone formation. Specimens implanted with HBS showed much less cement degradation and new bone formation than those implanted with the HBS blood composite (Figure 11). The HBS blood composite showed complete incorporation of the remaining cement particles into a new bone trabecular network. New bone formation was easily identified between and around the remaining calcium phosphate cement particles, as SEM imaging provided better definition than µ-CT to differentiate the remaining cement from the newly formed bone. As observed with µ-CT analysis, all tibias implanted with HBS blood composite always showed a more homogeneous bone healing response than HBS alone over the entire drilling groove.

**Figure 12:**
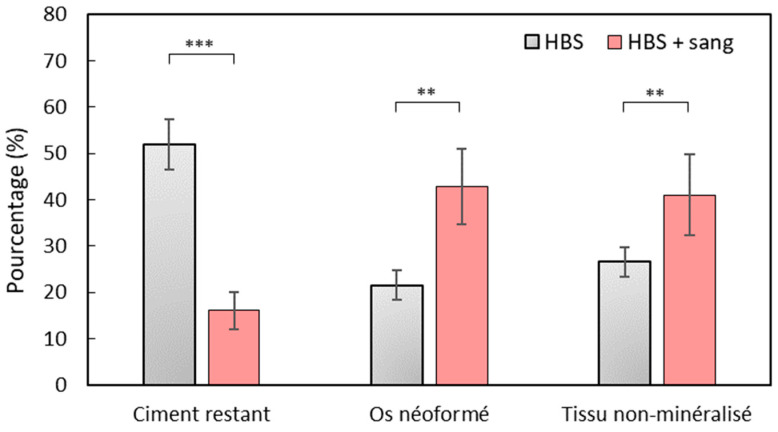
Quantitative SEM analysis after implantation of HBS vs. HBS ovine citrate blood composite (**: *p* < 0.01; ***: *p* < 0.001). Results are given as mean ± SD.

**Figure 13:**
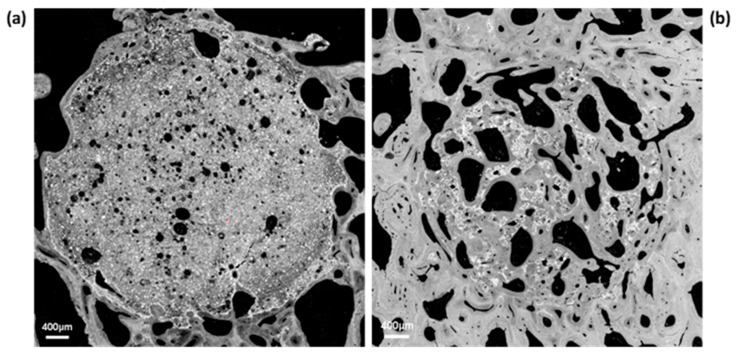
Quantitative SEM analysis after implantation of HBS vs. HBS blood composite. High magnification SEM images in transverse section of the bone tunnel, 4 months after implantation of HBS (**a**) vs. the HBS blood composite (**b**). Note the higher resorption/bone substitution process in the case of the HBS blood composite and the formation of a new trabecular bone network in which the remaining cement particles were embedded.

Quantitative image analysis of the SEM images allowed the determination of the amount of remaining cement by selecting an area centered on the bone tunnel, and cropped to remove the peripheral host bone. The remaining cement percentage in the tunnel filled with the HBS cement was 51.9 ± 12.3% vs. 16.1 ± 9.0% in the tunnel filled with the HBS blood composite (Figure 12). However, grey level thresholding remained difficult, and the percentage of remaining cement was probably underestimated, as seen in Figure 13.

SEM quantitative image analysis demonstrated a significant difference between HBS and the HBS blood composite regarding percentages of remaining cement (*p* < 0.001) and newly formed bone (*p* < 0.01).

### 3.8. Light Microscopy Histological Analyses

**Figure 14:**
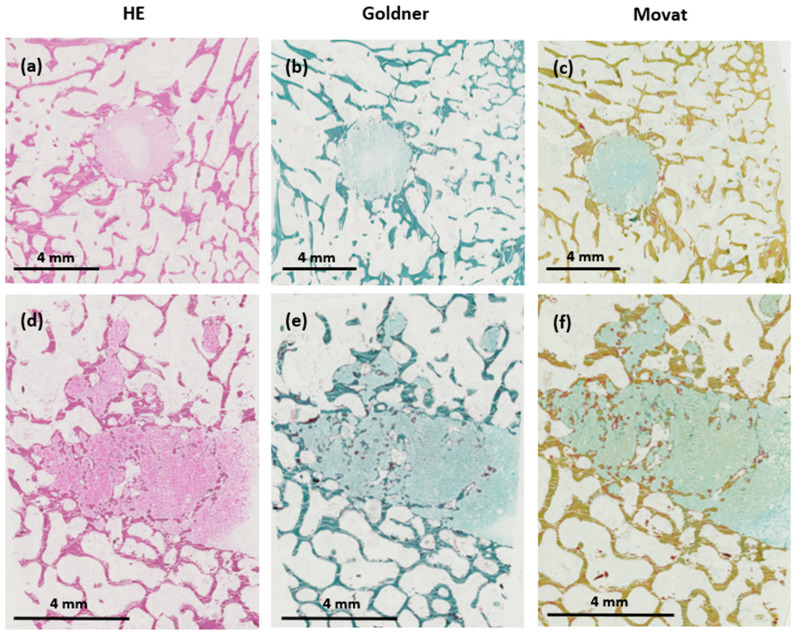
Histological images in transverse (**a**–**c**) and longitudinal sections (**d**–**f**) of the bone tunnel 4 months after implantation of the HBS cement after staining with hematoxylin–eosin (HE), Goldner’s trichrome, and Movat’s pentachrome, respectively.

**Figure 15:**
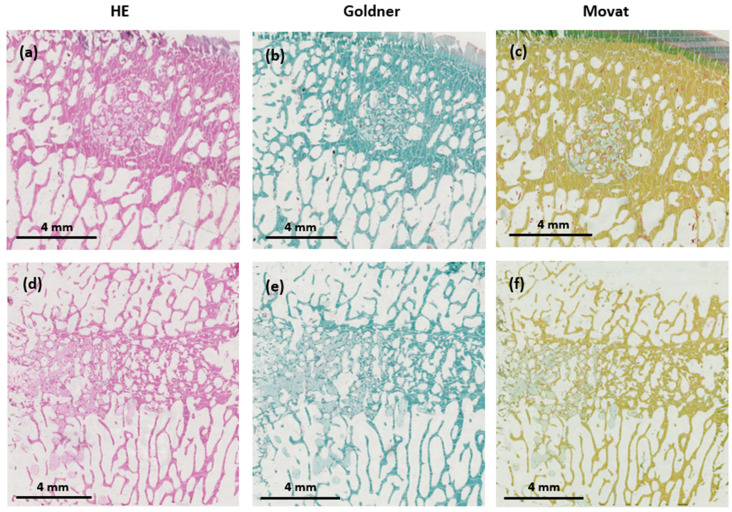
Histological images in transverse (**a**–**c**) and longitudinal sections (**d**–**f**) of the bone tunnel 4 months after implantation of the HBS ovine citrate blood composite after staining with hematoxylin–eosin (HE) staining, Goldner’s trichrome, and Movat’s pentachrome, respectively.

No adverse cellular effects were observed with any of the two injected cements (Figure 14 and Figure 15). Goldner’s trichrome and Movat’s pentachrome stains confirmed the important resorption of the HBS blood composite and its substitution by a newly formed bone trabecular network, whose mineralization and architecture were close to those present in the surrounding trabecular bone. Conversely, most of the bone defect was still filled with the HBS cement, which exhibited limited resorption and new bone formation.

Thanks to a more obvious color differentiation, Movat’s pentachrome stained sections were used to determine the respective amount of newly formed bone and remaining cement into a selected area of the bone tunnel. The results were obtained in pixel number distribution between bone and the remaining cement and transformed into percentages.

**Figure 16:**
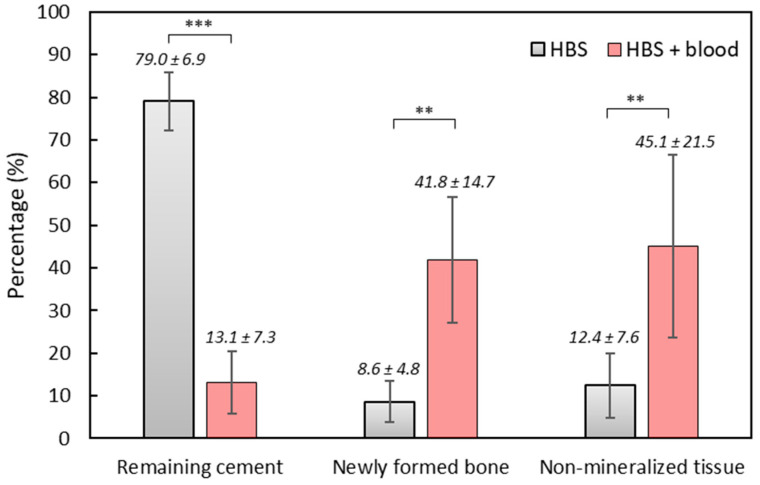
Quantitative light microscopy analysis after implantation of HBS vs. the HBS ovine citrate blood composite (**: *p* < 0.01; ***: *p* < 0.001). Results are given as mean ± SD.

Significant differences (*p* < 0.001) between HBS and the HBS blood composite were observed in the relative amount of new bone formation and cement resorption. In the bone cavities filled with HBS, the amount of newly formed bone was 8.58 ± 4.83% and the remaining cement amount was 79.03 ± 6.87%. In the bone cavities filled with the HBS blood composite, the amounts of newly formed bone and remaining cement were 41.82 ± 14.68 and 13.07 ± 7.31%, respectively (Figure 16).

The results were consistent with those observed in SEM for both cements, confirming a significantly greater resorption of the cement and higher new trabecular bone network formation after injection of the HBS blood composite. Quantitative analysis on stained sections appeared more accurate as the stained sections provided better distinction between the implanted cements and the new bone tissue than the grey level distribution of the SEM images.

It is important to note that the injection of calcium phosphate cements has been proposed as a viable option to treat BML, provided that a highly injectable and reactive cement can be injected into the lesion area. Indeed, as reported recently [39], the use of poorly reactive CPC having a slow resorbing rate was found not to be suitable for this indication in human patients, since no bone replacement was observed after a few years. As demonstrated by the in vivo results presented in this study, by contrast with the commercial HBS+ which showed very slow resorption, the parent blood composite exhibited a high resorption rate and bone replacement, which might thus make it a potentially interesting candidate for the treatment of BML. In fact, the homogeneous bone healing observed over the entire length of the drilling 4 months after implantation suggested that any long-term encapsulation of the implanted cement (HBS blood) might be unlikely, thus avoiding the release of CPC microparticles that would result in a chronic inflammation deleterious for the BML treatment [40].

## 4. Conclusions

A combination of the solid phase of commercial calcium phosphate (Graftys^®^ HBS) with blood resulted in an increase in its injectability properties (ca. decrease in the applied forces to inject the cement paste, and an improvement in the cohesion). Moreover, the use of blood as the liquid phase led to a significant increase in the setting time by ca. 7–15 h, depending on the blood source (ovine vs. human) and blood stabilizer (citrate vs. heparin). The granulometry of the cement powder was found to strongly influence the setting reaction since a decrease in the size of the cement particles resulted in a fast setting time. The slow formation of a 3D organic network was found to occur in the HBS blood composite. This phenomenon was found to affect the formation of CDA crystals in the intergranular space, leading to the presence of areas of low mineral density spread in the whole volume of the HBS blood composite.

When injected in tibial subchondral cancellous bone in a bone marrow lesion ovine model, quantitative SEM and histological analyses showed a highly significant difference between the HBS reference versus its analogue combined with blood. After a 4-month implantation, the cement resorption and new bone formation were remarkably high for the HBS blood composite, resulting in a very low amount of remaining cement and a high amount of newly formed bone in the treated area, while resorption was found to be very limited in the case of the HBS reference. These results demonstrated that the HBS blood composite was significantly more efficient than HBS to restore the trabecular architecture of the newly formed bone network. Given that the use of CPC for intra-osseous injection to treat BML has been shown to be of limited interest in the case of CPC having a slow resorption rate, the higher reactivity and resorbability of the HBS blood composite developed in this study might make it an interesting and relevant candidate for subchondroplasty.

## Figures and Tables

**Table 1:** Particle size parameters of the HBS, HBS-g26, and QS cement powders (analyses were performed in volume mode).

Parameters	HBS	HBS-g26	QS
Mean size (µm)	18.9	13.0	11.9
Median (µm)	45.3	53.9	52.1
Mode (µm)	18.7	5.21	4.58
d_10_ (µm)	1.65	0.87	0.88
d_50_ (µm)	9.86	5.92	6.17
d_90_ (µm)	35.3	31.1	47.7
Minimal size (µm)	0.11	0.11	0.11
Maximal size (µm)	272	272	111

**Table 2:** Dielectric permittivity and dielectric losses recorded at 37 °C during the hardening of HBS and HBS-g26 formulations and their analogues combined with blood (human or ovine) stabilized with heparin or citrate.

Studied Conditions	Dielectric Permittivity (ε′)		Dielectric Losses (ε″)	
	t_i_ (min)	t_f_ (min)	t_i_ (min)	t_f_ (min)
HBS	23	47	19	45
HBS + human citrated blood	620	740	610	790
HBS + human heparinized blood	450	583	450	670
HBS + ovine citrated blood	890	1050	860	1100
HBS + ovine heparinized blood	440	575	460	665
HBS-g26	<2	13	<2	14
HBS-g26 + human citrated blood	30	82	32	100
HBS-g26 + human heparinized blood	12	79	8	96
HBS-g26 + ovine citrated blood	30	88	32	113
HBS-g26 + ovine heparinized blood	33	99	34	123

**Table 3:** Monitoring of the kinetics of the blood clot formation, by decalcification of hardened HBS (left views), and HBS-g26 (right views) combined with ovine citrate blood.

Hardening Time at 37 °C (h)	HBS + Blood		Hardening Time at 37 °C (h)	HBS-g26 + Blood	
	Clotting	Pictures		Clotting	Pictures
12 h	No	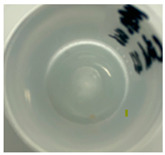	18 h	In progress	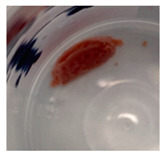
24 h	No	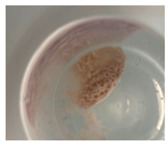	24 h	Yes	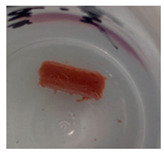
48 h	In progress	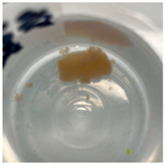	32 h	Yes	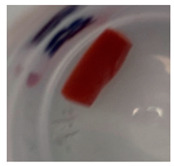
72 h	In progress	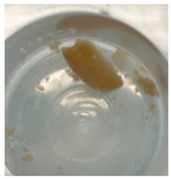	40 h	Yes	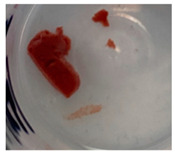
108 h	Yes	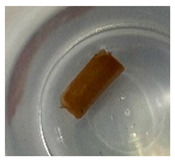	56 h	Yes	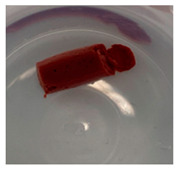
144 h	Yes	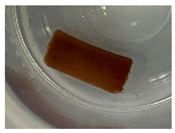	72 h	Yes	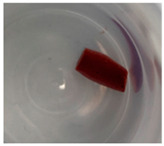

**Table 4:** Micro-CT quantitative measurement (ovine citrate blood was used for the HBS blood composite). Results are given as mean ± SD.

Formulations	BV/TV (%)	Trabecular Thickness (Tb.Th, mm)	Trabeculae Number per mm² (Tb.N)	Trabecular Separation (Tb.Sp, mm)
HBS	75.43 ± 7.91	0.88 ± 0.12	0.89 ± 0.10	0.51 ± 0.41
HBS blood composite	68.31 ± 11.98	0.46 ± 0.16	1.60 ± 0.27	0.34 ± 0.10
Control bone	45.26 ± 9.93	0.28 ± 0.15	1.60 ± 0.19	0.44 ± 0.09

## Data Availability

The data presented in this study are available on request from the corresponding author.

## Supplementary Materials

The following supporting information can be downloaded at https://www.mdpi.com/article/10.3390/jfb14040204/s1.
