# Supplementary material for: Calcium Phosphate Cements Combined with Blood as a Promising Tool for the Treatment of Bone Marrow Lesions
Source: J Funct Biomater. 2023 Apr 7;14(4):204. doi: 10.3390/jfb14040204 (PMC10143268; doi:10.3390/jfb14040204)
Supplement: Supplementary file 1 [file jfb-14-00204-s001.zip › jfb-2274345-supplementary.pdf]

Article

Supplementary Materials

Table S1. Quantitative SEM and light microscopy analysis of the percentage of remaining cement and newly formed bone after implantation of HBS *versus* HBS + blood composite.

Analyses	Remaining cement (%)	Newly formed bone (%)	Non-mineralized tissue (%)
SEM analysis			
HBS	51.91 ± 12.33	21.51 ± 7.15	26.59 ± 7.22
HBS + blood	16.11 ± 9.04	42.85 ± 18.4	41.05 ± 19.4
Light microscopy analysis			
HBS	79.03 ± 6.87	8.58 ± 4.83	12.39 ± 7.64
HBS + blood	13.07 ± 7.31	41.82 ± 14.68	45.11 ± 21.49

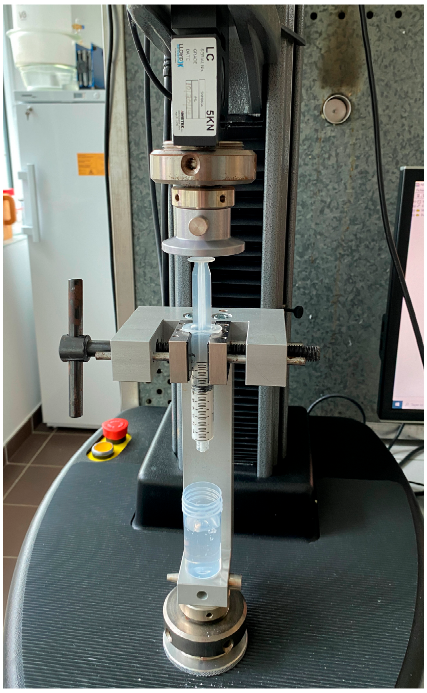

Figure S1. Injectability setup to measure the applied injection force in order to extrude the CPC paste through syringe without cannula.

Table S2. IRB certificate from harvested human blood (information provided by Cambridge Bioscience, UK).

Donor Information:

Blood Group	O RhD pos	HCV	Negative
Age / Gender	28 / M	Syphilis	Negative
Ethnicity	Black	HLA-A2 Antigen	*not tested*
HIV 1&2, & p24 Antigen	Negative	Reference Number	RDN-4110990
HBsAg	Negative		

Full Blood Count Indices (Pre-Donation):

WBC	6.18 $x10^9$/L	PLT	216 $x10^9$/L
RBC	5.76 $x10^{12}$/L	RDW	12.8 %
HGB	161 g/L	Neut	4.31 $x10^9$/L
HCT	0.513 L/L	Lymph	1.29 $x10^9$/L
MCV	89.1 fL	Mono	0.40 $x10^9$/L
MCH	28 pg	EO	0.16 $x10^9$/L
MCHC	314 g/L	Baso	0.02 $x10^9$/L
